# Prognostic value of CPSS cytogenetic risk classification in patients with CMML after allogeneic hematopoietic cell transplantation: a retrospective multicenter study of the Chronic Malignancies Working Party of the EBMT

**DOI:** 10.1038/s41409-022-01759-7

**Published:** 2022-07-23

**Authors:** Christian Koenecke, Dirk-Jan Eikema, Sheree Hazelaar, Thomas Schroeder, Victoria Potter, Nicolaus Kröger, Martin Bornhäuser, Jürgen Finke, Uwe Platzbecker, Aleksandar Radujkovic, Arnold Ganser, Urpu Salmenniem, Didier Blaise, Guido Kobbe, Ellen Meijer, Lone Friis, Johan Maertens, Dolores Caballero, Jan J. Cornelissen, Attilio Olivieri, Ozet Gulsum, Patrick J. Hayden, Francesco Onida, Marie Robin, Ibrahim Yakoub-Agha

**Affiliations:** 1 Hannover Medical School, Hannover, Germany; 2 EBMT statistical Unit, Leiden, The Netherlands; 3 EBMT data office Leiden, Leiden, The Netherlands; 4 Universitätsklinikum Essen, Essen, Germany; 5 GKT School of Medicine, London, UK; 6 University Hospital Eppendorf, Hamburg, Germany; 7 University Hospital TU Dresden, Dresden, Germany; 8 University of Freiburg, Freiburg, Germany; 9 University Hospital Leipzig, Leipzig, Germany; 10 University of Heidelberg, Heidelberg, Germany; 11 HUCH Comprehensive Cancer Center, Helsinki, Finland; 12 Institut Paoli-Calmettes, Marseille, France; 13 Heinrich Heine Universitaet, Duesseldorf, Germany; 14 Vrije Universiteit Medical center, Amsterdam, The Netherlands; 15 Rigshospitalet, Copenhagen, Denmark; 16 University Hospital Gasthuisberg, Leuven, Belgium; 17 Hospital Clinico, Salamanca, Spain; 18 Erasmus MC Cancer Institute, Rotterdam, The Netherlands; 19 Azienda Ospedali Riuniti di Ancona, Ancona, Italy; 20 Ankara Sehir Hastanesi, Ankara, Turkey; 21 Trinity College Dublin, St. James’s Hospital, Dublin, Ireland; 22 Fondazione IRCCS Ca’ Granda Ospedale Maggiore Policlinico-University of Milan, Milan, Italy; 23 Hopital St. Louis, Paris, France; 24 CHU de Lille, INSERM U1286, Infinite, Université de Lille, 59000 Lille, France

**Keywords:** Haematological diseases, Haematological cancer

## To the Editor:

The only curative treatment approach for patients with chronic myelomonocytic leukemia (CMML) is allogeneic hematopoietic cell transplantation (allo-HCT), but disease relapse after transplantation is a major concern [1]. Predictors for disease outcome after transplant are limited. However, beside other risk factors (ASXL1 mutations, monocytosis, cytopenias and circulating immature myeloid cells), cytogenetic abnormalities have been shown to serve as predictors for outcome in CMML patients [2–5]. Cytogenetic abnormalities are frequently seen in 20–30% of patients [6, 7]. According to the CMML-specific prognostic scoring system (CPSS) patients can be categorized into three risk groups (high risk: trisomy 8, chromosome 7 abnormalities, or complex karyotype; low risk: normal karyotype and -Y; intermediate risk: all other chromosomal abnormalities) [8]. There is evidence, that adverse cytogenetics are also a risk factor for worse outcome after allo-HCT [9]. However, cytogenetic information according to CPSS was not evaluated in the setting of allo-HCT to date. Therefore, the aim of this large multicentric, international study was to retrospectively determine the impact of CPSS-cytogenetic on outcome after allo-HSCT.

Adult patients (age > = 18years) who had received a first allo-HCT for the treatment of CMML between 2000 and 2015 were selected from the European Society of Bone and Marrow Transplantation (EBMT) database. 233 centers participated into this study. In total, 1347 patients were included. Impact of CPSS-cytogenetic classification was analysed regarding overall survival (OS), progression free survival (PFS) and cumulative incidence of relapse and non-relapse mortality (NRM) after transplant. OS and PFS were estimated using the Kaplan-Meier product limit estimation method, and differences in subgroups were assessed by the Log-Rank test. Median follow-up was determined using the reverse Kaplan-Meier method. The cumulative incidence of relapse (RI) and NRM were analysed together in a competing risks framework. Subgroup differences in cumulative incidences were assessed using Gray’s test. Multivariable Cox regression was applied to investigate the simultaneous impact of multiple covariates on OS and PFS. Included covariates were: CPSS (intermediate, high versus low), stage at transplant (no CR, untreated versus CR), disease (transformed to AML versus other), age at transplant (in decades) and year of allo-HCT. Continuous variables are presented in the text as median and interquartile range (IQR) or range and categorical variables as percentages. All survival estimates and hazard ratios are reported with corresponding 95% confidence intervals in parentheses. All *p*-values are two-sided and *p* < 0.05 is considered significant. Statistical analyses were performed in R version 3.6.0 (R Development Core Team, Vienna, Austria), using packages ‘survival’, ‘prodlim’ and ‘cmprsk’.

436 female (32.4%) and 909 male (67.6%) patients were included into the study. Median age at HSCT was 58.1 years (range 20–75.4). At time of HCT, 383 (68.6%) patients were diagnosed with CMML-I, 175 (31.4%) with CMML-II, 412 (74.9%) with dysplastic and 138 (25.1%) with proliferative CMML. Only 392 (30.6%) patients were in complete remission, whereas 668 (52.2%) had not reached CR and 220 (17.2%) had not received chemotherapy before allo-HCT. 212 (35.0%) patients received conventional chemotherapy and 119 (19.6%) hypomethylating agents before transplantation. Matched related donor allo-HCT was performed in 35.6% of the patients, matched unrelated donor in 7.4%, unrelated donor (complete HLA unavailable) in 51.6%, mismatched related in 2.8% and mismatched unrelated in 2.6%. Bone marrow (10.2%), peripheral blood (87.3%), or both (0.2%) served as the stem cell graft. Cord blood was used in 2.3%. Myeloablative conditioning regimens were used in 187 patients (13.9%), and less intensive regimens were given to 1156 patients (86.1%). Median follow-up of patients was 51.4 (47.8–56.8) months.

Two-year and five-year PFS were 39% (36–42%) and 29% (26–32%), respectively. Two- and 5-year relapse incidence were 35% (33–38%) and 41% (38–44%) respectively, with a relapse observed in 474 patients at any time during follow-up. The median time to relapse in the patients who relapsed was 4.9 months (IQR 2.7–11.7). Two- and 5-year NRM were 26% (23–28%) and 30% (27–33%), respectively. Two- and 5-year OS were 46% (43–49%) and 33% (30–36%).

**Fig. 1:**
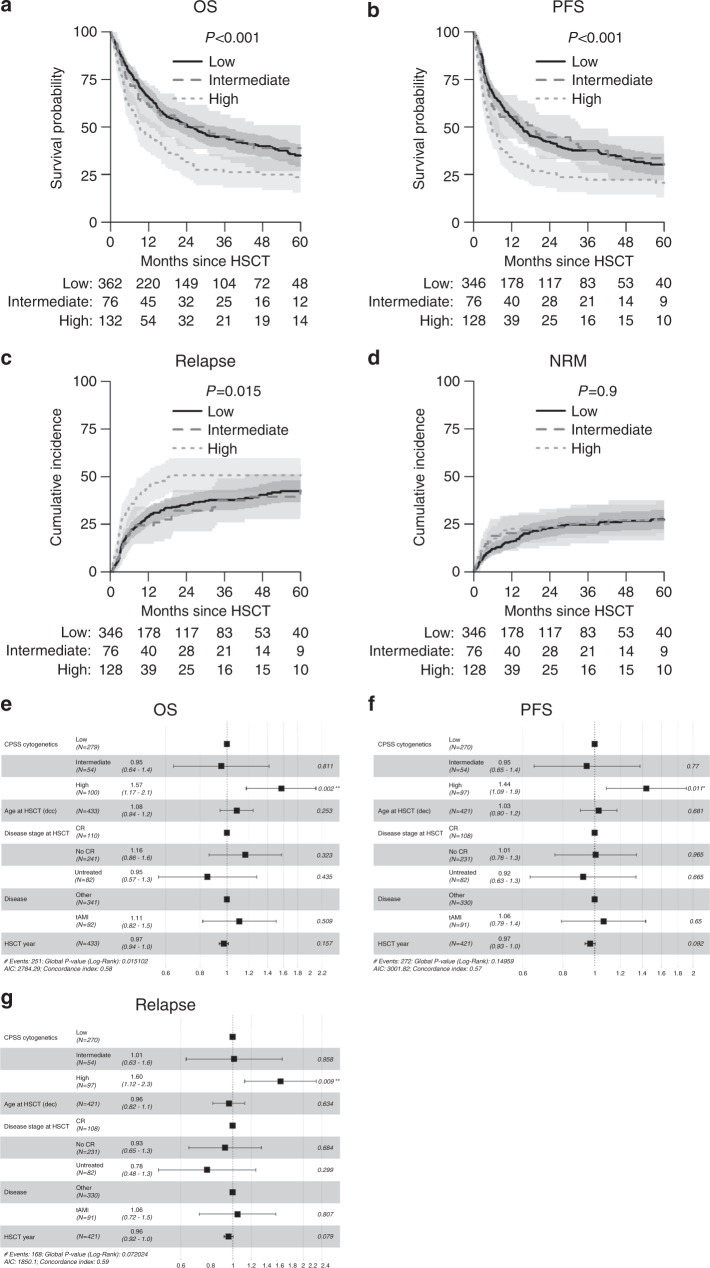
CPSS cytogenetic information predict outcome after allogeneic transplantation in CMML patients. **a**–**d** Probability of overall survival (OS), progression free survival (PFS), relapse incidence and non relapse mortality (NRM) of CMML patients with low, intermediate or high risk cytogentic. **e**–**g** Forest plots on overall survival (OS), progression free survival (PFS) and relapse incidence.

570 patients had sufficient cytogenetic information according to CPSS (777 missing). 132 (23.2%) patients could be categorized into CPSS-high, 76 (13.3%) into intermediate and 362 (63.5%) into low-risk cytogenetics, respectively. In univariate analysis CPSS cytogenetic information was found to be strongly associated with OS (*p* < 0.001; low 35% (29–41%), intermediate 39% (27–51%), high 24% (15–32%)) at 5 years. A higher cumulative incidence of relapse (*p* = 0.015; low 42% (37–48%), intermediate 43% (30–56%), high 51% (42–60%)) was detected. In line, cytogenetic status was associated with PFS (*p* < 0.001; low 30% (25–36%), intermediate 30% (18–42%), high 21% (13–28%). However, NRM (*p* = 0.87; low 27% (22–33%), intermediate 27% (16–38%), high 29% (20–37%)) at 60 months was not affected by cytogenetic status at time of transplantation (Fig. 1).

In multivariable analysis (MVA), including only patients with available data on included covariates, CPSS-high risk cytogenetics was associated with shorter overall survival after allogeneic transplantation compared to intermediate and low risk cytogenetics (hazard ratio (HR), 1.57 (1.17–2.1)). This finding was also present in MVA for PFS (HR 1.44 (1.09–1.91)) and RI (HR 1.6 (1.12–2.27)). Patients’ age, year of transplant, and status of the disease at transplant were not associated with a reduced OS, PFS and RI (Table 1). In another study by this working group, status of the disease before transplantation was associated with survival [10].

**Table 1:** Multivariate analysis overall (OS), progression free survival (PFS) and relapse incidence (RI).

		OS			PFS			RI		
		*N*	HR (95% CI)	*p*	*N*	HR (95% CI)	*p*	*N*	HR (95% CI)	*P*
	Total	433			421			421		
CPSS	Low	279			270			270		
	Intermediate	54	0.95 (0.64–1.42)	0.8	54	0.95 (0.65–1.37)	0.8	54	1.01 (0.63–1.62)	>0.99
	High	100	1.57 (1.17–2.1)	0.002	97	1.44 (1.09–1.91)	0.01	97	1.6 (1.12–2.27)	0.009
Stage at transplant	CR	110			108			108		
	no CR	241	1.16 (0.86–1.58)	0.3	231	1.01 (0.76–1.34)	0.9	231	0.93 (0.65–1.33)	0.7
	Untreated	82	0.85 (0.57–1.28)	0.4	82	0.92 (0.63–1.34)	0.7	82	0.78 (0.48–1.25)	0.3
Disease	Other	341			330			330		
	tAML	92	1.11 (0.82–1.5)	0.5	91	1.06 (0.79–1.43)	0.7	91	1.05 (0.72–1.52)	0.8
Age (decades)		433	1.08 (0.94–1.24)	0.3	421	1.03 (0.9–1.17)	0.7	421	0.96 (0.82–1.13)	0.6
Tx year		433	0.97 (0.94–1.01)	0.16	421	0.97 (0.93–1.01)	0.09	421	0.96 (0.92–1)	0.08

Our results show that CPSS cytogenetics is a strong predictor of relapse and overall survival after allo-HCT. Adverse cytogenetic alterations lead to a disease biology which is more likely to be resistant to an allograft. This observation has been made in a variety of myeloid malignant diseases, such as MDS and AML [11]. Currently, molecular diagnostics are becoming more and more standard in patients with CMML. We were not able to include such information into this retrospective study. It might be that even more distinct risk-groups can be identified using that diagnostic tool [5].

In this international, multicentric analysis we show that CMML patients with high-risk cytogenetics had significantly worse overall and progression-free survival after allo-HCT than patients with intermediate or low risk cytogenetics according to CPSS. New therapeutic strategies to prevent relapse after allo-HCT in CMML patients with high-risk cytogenetics are needed.

## Data Availability

CK, DJE, and IYA had full access to all study data (available upon data-specific request).
